# Lower-Limb Sensorimotor Deprivation-Related Brain Activation in Patients With Chronic Complete Spinal Cord Injury

**DOI:** 10.3389/fneur.2020.555733

**Published:** 2020-10-06

**Authors:** Feng Gao, Yun Guo, Hongyu Chu, Weiyong Yu, Zhenbo Chen, Liang Chen, Jun Li, Degang Yang, Mingliang Yang, Liangjie Du, Jianjun Li, Chetwyn C. H. Chan

**Affiliations:** ^1^School of Rehabilitation Medicine, Capital Medical University, Beijing, China; ^2^Department of Spinal and Neural Functional Reconstruction, China Rehabilitation Research Center, Beijing, China; ^3^SCI Unit, China Rehabilitation Science Institute, Beijing, China; ^4^Center of Neural Injury and Repair, Beijing Institute for Brain Disorders, Beijing, China; ^5^Beijing Key Laboratory of Neural Injury and Rehabilitation, Beijing, China; ^6^Department of Rehabilitation Medicine, School of Clinical Medicine, Beijing Tsinghua Changgung Hospital, Tsinghua University, Beijing, China; ^7^Department of Comprehensive Rehabilitation, China Rehabilitation Research Center, Beijing, China; ^8^Department of Radiology, China Rehabilitation Research Center, Beijing, China; ^9^Applied Cognitive Neuroscience Laboratory, Department of Rehabilitation Sciences, The Hong Kong Polytechnic University, Hong Kong, China

**Keywords:** compensatory strategies, motor imagery, functional imaging, functional reorganization, spinal cord injury

## Abstract

This study aims to investigate functional brain reorganization brought about by the loss of physical movement and sensory feedback in lower limbs in chronic spinal cord injury (SCI). Eleven paraplegia patients with SCI and 13 healthy controls (HCs) were recruited. The experimental task used was a visuomotor imagery task requiring subjects to engage in visualization of repetitive tapping movements of the upper or lower limbs. Blood oxygen level-dependent (BOLD) responses were captured during the experimental task, along with the accuracy rate and the response time. The SCI patients performed worse in the Rey Auditory Verbal Learning Test (RAVLT) and the Trail Making Test. SCI patients had a larger BOLD signal in the left lingual gyrus and right external globus pallidus (GPe) when imagining lower-limb movements. For the upper-limb task, SCI patients showed stronger BOLD responses than the HCs in extensive areas over the brain, including the bilateral precentral gyrus (preCG), bilateral inferior parietal gyrus, right GPe, right thalamus, left postcentral gyrus, and right superior temporal gyrus. In contrast, the HCs displayed stronger BOLD responses in the medial frontal gyrus and anterior cingulate gyrus for both upper- and lower-limb tasks than the SCI patients. In the SCI group, for the upper-limb condition, the amplitudes of BOLD responses in the left preCG were negatively correlated with the time since injury (*r* = −0.72, *p* = 0.012). For the lower-limb condition, the amplitudes of BOLD responses in the left lingual gyrus were negatively correlated with the scores on the Short Delay task of the RAVLT (*r* = −0.73, *p* = 0.011). Our study provided imaging evidence for abnormal changes in brain function and worsened cognitive test performance in SCI patients. These findings suggested possible compensatory strategies adopted by the SCI patients for the loss of sensorimotor function from the lower limbs when performing a limb imagery task.

## Introduction

Traumatic spinal cord injury (SCI) is a devastating neurological disorder that affects sensory and motor function below the level of injury due to disconnected ascending and descending tracts (1, 2). Management of traumatic SCI is challenging. In most cases, surgical intervention is required. Moreover, rehabilitation is crucial for its functional recovery. Several studies have reported the potential role of motor imagery (MI) in motor performance for healthy subjects (3, 4), and in motor recovery for SCI patients (5, 6). Although MI is a kind of mental rehearsal of a motor act without any overt movement execution (ME) (7), it is believed that MI and ME rely on similar neural mechanisms (7). In view of the overlapping networks with the motor preparation process, MI is considered crucial for investigating the motor control system, especially in the stage of motor preparation, motor planning, or motor programming in these individuals (6).

Previous functional MRI studies found that brain activation in SCI patients undergoing MI is regulated by sensorimotor stimulation (8–13). Yet, so far, only a few neuroimaging studies have examined the effect of MI on upper and lower limbs in thoracic SCI. These patients have normal functions of upper limbs but lose their ability to control the lower limbs. A previous study showed that the primary somatosensory cortex representations of the thumb and little finger were medially displaced in complete thoracic SCI patients compared to controls (13). Another research indicated that increased use of upper limbs by SCI patients suffering from paralyzed lower limbs could cause sensorimotor activation in the brain (14). Yet, it remains unclear whether sensorimotor activation may be different for lower and upper limbs in paralyzed patients with SCI. Moreover, MI is a cognitive process; nevertheless, so far, only a few studies have evaluated the cognitive performance in patients with SCI.

The aim of this study was to investigate functional brain reorganization and cognitive behavior in SCI patients with complete paralysis below the thoracic level using functional MRI. Herein, the repetitive upper and lower MI tasks were used. We hypothesized that functional brain activation in upper and lower limbs would be different between SCI patients and healthy controls (HCs).

## Materials and Methods

### Subjects

Traumatic SCI patients with American Spinal Injury Association (ASIA) Impairment Scale Grade A (complete SCI) (15), time post-injury >6 months, injury level from T7 to T12, age <60 years, and no additional neurological diagnoses were invited from the China Rehabilitation Research Center (CRRC) in Beijing. HCs were matched based on age, gender, education, the time point of scanning, and neurological health. All the subjects were right hand and foot dominant, had no contraindications to MRI, did not have metals implanted in the body, had no neuropathic pain, were not pregnant, and were not using medications that lower seizure threshold such as tricyclic antidepressants or neuroleptics. Dr. Gao and Dr. Yu assessed the SCI subjects and HCs. The SCI subjects were asked to attend a clinical interview and physical examination to determine the level and completeness of injury. HCs had no illness history, had a normal Mini-Mental State Exam assessment, and underwent cranial MRI scanning.

The procedures in this study were approved by the Ethics Committee on Human Studies of CRRC and the Departmental Ethics Committee of the Department of Rehabilitation Sciences, the Hong Kong Polytechnic University (2011-017). Each subject was asked to sign a written informed consent form that included the description of the study, the risk of fMRI scanning, study procedures, and the transportation tool before participation in the study. Each subject was assigned a code that was utilized throughout the study, including the training, scanning, data processing, and analysis.

### Study Design

**Figure 1:**
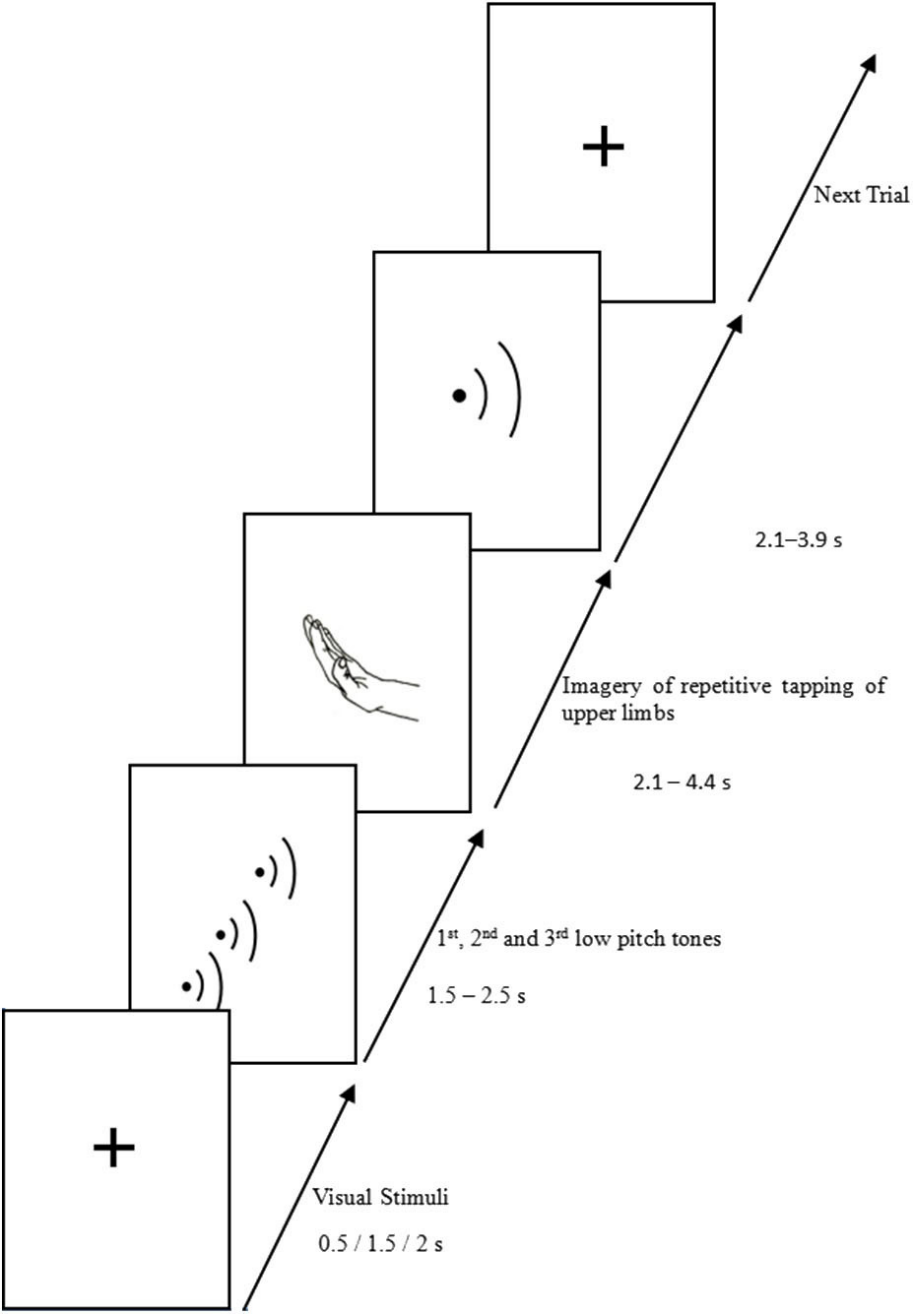
Summary of the paradigm of the motor imagery task (take the upper limb as example).

The experimental paradigm used for functional brain imaging was a repetitive movement imagery task, herein called the MI task. There were two conditions in the MI task, upper vs. lower limbs. All the movements were rhythmic and alternated, involving the right and left limbs. The participants were asked to keep pace with the internally generated rhythm and to imagine the right and left limbs tapping alternatively on a table or on the floor according to the rhythm. The tapping movements had three rhythmic patterns of 0.8, 1.0, and 1.33 Hz (16). A typical trial began with three auditory cues presented at one of the three rhythmic patterns (Figure 1). The subject followed the rhythm of the tones and began visualizing the tapping movement of upper or lower limbs, one after the other, for 2.1–4.4 s. After hearing a high-pitched tone, the subject stopped to visualize movements and indicated which side of the limb was toward the platform at that instance by pressing a button on a keyboard. The duration of capturing BOLD responses by the scanner was 2.0 s, beginning from the presentation of the third auditory cue. Accuracy rate (ACC) and mean response time (RT) were the behavioral parameters of the task.

### Procedures

A telephone interview was first used to screen potential subjects. Consequently, each was registered on the notice board and then evaluated by a senior clinical doctor, who evaluated the patient general physical examination, visual and auditory testing, general cognitive measures, handedness and dominant foot, and a specialized neurological examination for SCI subjects. Eligible subjects were confirmed with the inclusion and exclusion criteria.

When the subjects decided to participate in this study, a tentative date for the fMRI scan was scheduled. Two days before the scan, the training session began, and the subjects received standardized training described as the “Training session” to assure the accuracy in completing at least 70% of the task.

The subjects were transported to Beijing Xuanwu Hospital to carry out the fMRI scanning. On the same day of the scan, they were asked to complete the neuropsychological tests with a certified psychologist, including the Digit Span Forward Test, the Digit Span Backward Test, the Rey Auditory Verbal Learning Test (RAVLT)–Chinese Version, the Symbol Digit Modalities Test (SDMT), the Trail Making Test (TMT) A & B–Chinese Version, and the Stroop Test–Chinese Version (17). All subjects were asked to avoid caffeine or alcohol for at least 12 h prior to behavioral testing or fMRI scanning.

### fMRI

All fMRI scans were conducted at Beijing Xuanwu Hospital on a 3-Tesla scanner (Siemens Medical Solutions, Germany). Auditory stimuli were induced through earplugs and headphones, which were also used to avoid the noise of the MRI machine, and to enable communication. Briefly, the subject's head was placed on surrounding foam cushions to restrict movement throughout the scanning. Visual Basic (Visual Studio 2005 version) installed on a standard RRI computer was used to present the visual stimuli. The visual stimuli were projected on a screen in front of the scanner and seen by the subject through a mirror attached to the head coil. The exact spot, the angle, and the height of the screen were adjusted and marked in the floor to ensure the same conditions of the visual stimuli for all subjects.

BOLD functional images were acquired using T2^*^-weighted gradient echo planar imaging (EPI) sequences covering 32 slices (3 mm thick, zero gap), aligned on the axial-oblique plane, and encompassing the entire cerebral cortex. The parameters for functional images were the following: TR = 2,000 ms, TE = 30 ms, FOV = 220 × 220 mm^2^, flip angle = 90°, voxel size = 3.4 × 3.4 × 3.0 mm, and interleaved acquisition. The parameters for structural images were as follows: TR = 1,600 ms, TE = 2.13 ms, FOV = 256 × 256, flip angle = 90°, slice thickness = 1 mm (no gap), voxel size = 1.0 × 1.0 × 1.0 mm, and interleaved acquisition.

### Data Analysis

Both the accuracy rate and response time of the repetitive and daily tasks were recorded. The comparison of the two factors, i.e., group (chronic SCI vs. HC) and limbs (upper limb vs. lower limb), was performed using repeated ANOVA in SPSS 20.0.

All preprocessing of fMRI images was conducted using SPM8 (www.fil.ion.ucl.ac.uk/spm). To further reduce the effects of confounding factors, linear regression was used to further remove the effects of head motion and other possible sources of artifacts. An uncorrected voxel-level intensity threshold of *p* < 0.001 with a minimum cluster size of 35 contiguous voxels was used to correct for multiple comparisons using the AlphaSim method (http://afni.nimh.nih.gov/pub/dist/doc/manual/AlphaSim.pdf). This process yielded a corrected threshold of *P* < 0.05. The time post-injury was tested as a covariate in fMRI analysis.

A secondary analysis of the regional BOLD time-series signal within the general linear model was conducted to estimate the effects of task presentation on the regions of interest (ROIs). The ROIs were defined as the activated clusters in each anatomical neural substrate based on the previous results of the whole-brain analysis. Each valid event of the imagery task with correct responses was assumed to produce a response lasting 10 time points (~18-s response epoch) after the performance of the imagery task, in which the time course of BOLD responses per ROI was computed as the expected hemodynamic response function (HRF) reflecting the real neural activities of the most reliable index (17). No assumptions were made about the shape of the response at this stage of analysis.

Statistical analysis was performed using SPSS software, version 20.0. Continuous variables were tested using two-tailed *t*-tests, while chi-square tests examined gender differences. *P* < 0.05 was considered to be statistically significant. For both SCI and HC groups, Spearman correlation coefficients were calculated between the magnitude of the MR signal of the ROIs, the significant differences in the clinical measure, the behavioral results during the experimental task, and the specific clinical data for the SCI group, such as the time since injury.

## Results

### Demographic Characteristics

A total of 11 SCI patients and 13 HCs were included in the analysis. The demographic characteristics of SCI subjects are presented in Table 1. The time post-injury in the SCI group varied from 9 to 172 months (64.9 ± 47.4 months). There were no differences in age, the mean year of education, and the proportions of gender between the HC and SCI groups.

**Table 1:** Clinical data, functional measures, and neurological grades using the American Spinal Injury Association Impairment scale (ASIA) of SCI patients.

**No**.	**Age**	**Sex**	**Etiology**	**Injury level**	**ASIA**	**Time post injury (months)**	**VAS**	**SCIM**
01	37	M	Fall	T11	A	9	0	71
02	38	M	Fall	T10	A	12	0	65
03	41	F	Traffic accident	T11	A	13	0	60
04	23	F	Fall	T10	A	41	0	65
05	16	F	Traffic accident	T7	A	67	0	56
06	55	M	Fall	T10	A	79	0	52
07	40	F	Heavy pound	T8	A	97	0	58
08	57	M	Traffic accident	T7	A	172	0	53
09	32	M	Fall	T10	A	63	0	70
10	36	M	Fall	T8	A	76	0	63
11	33	F	Heavy pound	T10	A	85	0	65

*SCI, spinal cord injury; HCs, healthy controls; VAS, Visual Analog Scale; SCIM, Spinal Cord Independence Measure*.

### Behavioral Results From Cognitive Tests

Between-group differences on cognitive tests were revealed in two tests (Table 2). For RAVLT, SCI patients scored significantly lower on the Short Delay task than those in HCs (*t* = 2.11, *p* = 0.04). SCI patients also scored significantly lower on the RAVLT (Recognition) than their HC counterparts (*t* = 2.302, *p* = 0.03). SCI patients performed worse than those in the HCs (*t* = −2.29, *p* = 0.04), as reflected by the TMT difference scores (i.e., B minus A).

**Table 2:** Performances on cognitive tests of subjects in the SCI and HC groups.

	**SCI group mean (SD)**	**HC group mean (SD)**	***t*-value**	***p*-value**
**Digit span test**				
Forward	10.6 (2.1)	11.2 (2.9)	0.562	0.58
Backward	5.1 (3.4)	6.8 (4.0)	1.103	0.28
**RAVLT**				
Short delay	29.5 (3.1)	33.3 (5.2)	2.108	0.04^*^
Long delay	12.3 (1.8)	12.8 (2.0)	0.735	0.47
Recognition	25.3 (3.4)	28.0 (1.9)	2.302	0.03^*^
SDMT	46.0 (11.1)	54.3 (12.4)	1.718	0.10
**TMT**				
A (s)	41.5 (15.6)	32.7 (17.1)	−1.296	0.21
B (s)	91.8 (50.8)	54.9 (31.6)	−2.091	0.05
B-A (s)	50.3 (36.2)	22.1 (20.3)	−2.292	0.04^*^
**Stroop test^#^**				
Word	0.547 (0.130)	0.454 (0.166)	−1.493	0.15
Color	0.715 (0.119)	0.675 (0.237)	−0.526	0.61
Word-color	1.118 (0.169)	1.03 (0.245)	−1.015	0.32

# *In the Stroop test, the scores were computed as composite quotients by dividing the time required for completing the test by the number of correct responses*.

* *p < 0.05*.

*RAVLT, rey auditory verbal learning test; SDMT, symbol digit modalities test; TMT, trail making test*.

### Behavioral Results on the Experimental Tasks

The subjects performed the MI tasks inside the scanner. Performance was expressed in terms of accuracy rate and response time. A 2 × 2 repeated-measure ANOVA with Lower Limb vs. Upper Limb as Condition and SCI vs. HCs as Group was conducted to test the differences in task performance, including ACC, RT, and their composite quotients (Table 3). The composite quotients were computed by dividing the ACC by the RT, called the ACC/RT. The results showed that Condition had no significant effect on subjects' ACC [*F*_1, 22_ = 0.46, *p* = 0.51], RT [*F*_1, 22_ = 3.86, *p* = 0.06], or ACC/RT [*F*_1, 22_ = 1.37, *p* = 0.25]. Likewise, Group showed no significant effect on subjects' ACC [*F*_1, 22_ = 0.12, *p* = 0.73], RT [*F*_1, 22_ = 0.24, *p* = 0.63], or ACC/RT [*F*_1, 22_ = 0.25, *p* = 0.62]. The Condition × Group interaction also showed no statistically significant effect on ACC [*F*_1, 22_ = 1.35, *p* = 0.26], RT [*F*_1, 22_ = 0.03, *p* = 0.87], or ACC/RT [*F*_1, 22_ = 0.48, *p* = 0.49].

**Table 3:** ACC and RT of subjects in the SCI and HC groups on the motor imagery tasks.

	**SCI (*n* = 11)**	**Healthy control (*n* = 13)**
**ACC (%)**		
Upper-limb motor imagery	45.7 (14.2)	46.8 (10.2)
Lower-limb motor imagery	49.7 (12.8)	45.7 (9.7)
**RT (ms)**		
Upper-limb motor imagery	1069.6 (358.7)	1135.2 (350.9)
Lower-limb motor imagery	993.6 (295.3)	1045.3 (236.2)
**ACC/RT**		
Upper-limb motor imagery	0.052 (0.012)	0.046 (0.017)
Lower-limb motor imagery	0.055 (0.022)	0.055 (0.023)

*SCI, spinal cord injury; ACC, accuracy rate; RT, response time*.

### Brain Imaging–Whole-Brain Analysis Between Two Groups

**Figure 2:**
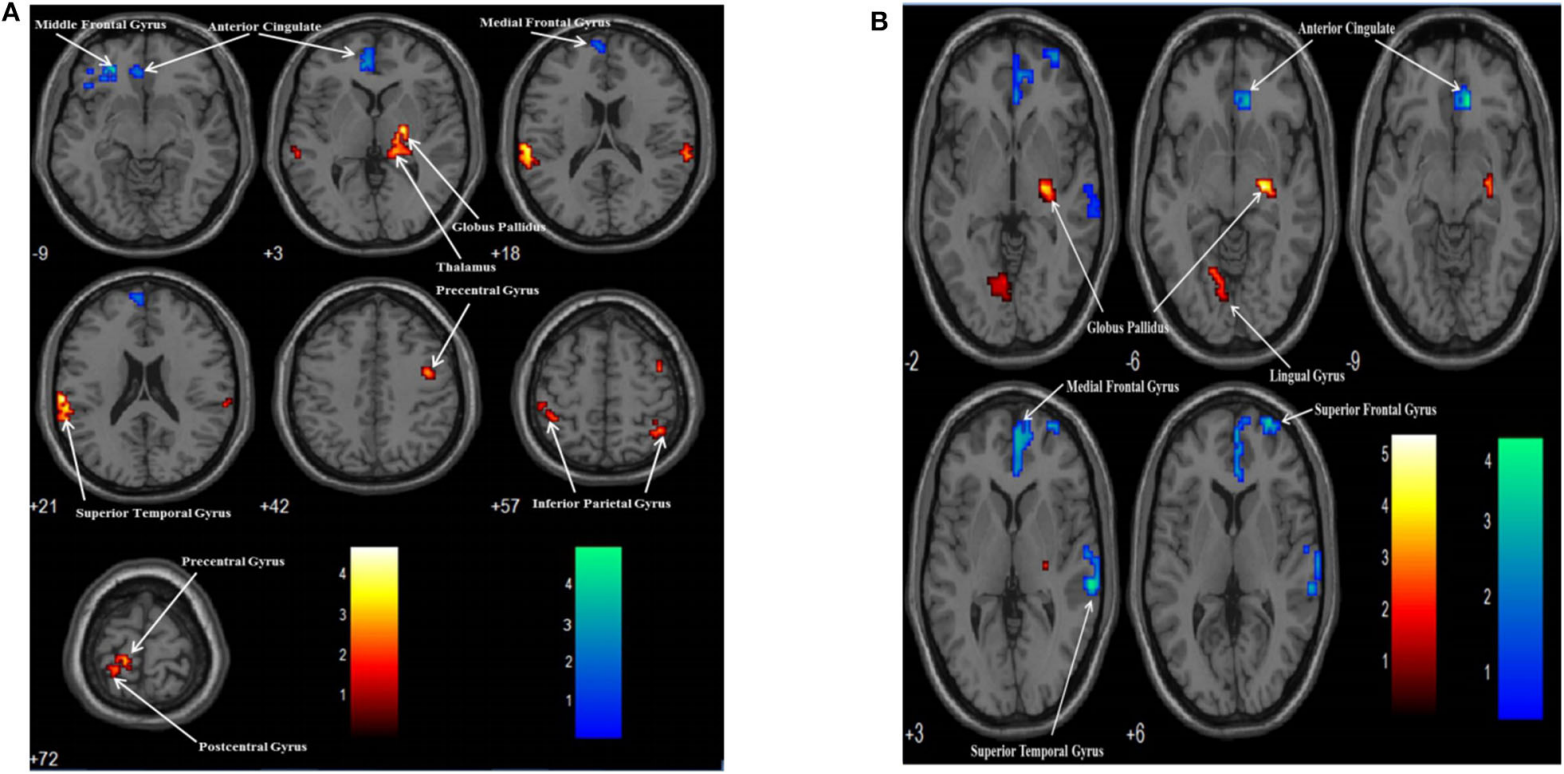
Group contrasts of BOLD responses between the SCI and HC groups when performing the upper limb **(A)** and lower limb **(B)** motor imagery task. Warm color: SCI vs. HC; Cool color: HC vs. SCI.

There were two group contrasts conducted for the BOLD responses elicited during the upper-limb condition. For the HC vs. SCI group contrast, the HC group showed significantly stronger BOLD responses than the SCI group in the left middle frontal gyrus (MiFG), left anterior cingulate (ACG), and left medial frontal gyrus (MeFG) (Table 4, Figure 2A). For the SCI vs. HC contrast, the results revealed significantly stronger BOLD responses in the SCI group than the HC group in the left inferior parietal gyrus (IPG), left precentral gyrus (preCG), and left postcentral gyrus (posCG). A stronger BOLD response in the SCI group was also revealed in the right globus pallidus (GP), right thalamus, right superior temporal gyrus (STG), right IPG, and right preCG. Activation in the right GP was further located in the lateral aspect of the neural substrate, which is called the external globus pallidus or globus pallidus external segment (GPe), and those in the right thalamus were located in the right pulvinar.

**Table 4:** BOLD responses elicited in the upper and lower limbs condition of the motor imagery tasks between subjects in the SCI and HC groups (cluster size ≥35, *p* < 0.01 with multiple corrections).

**Conditions**	**Cluster**	**Label**	**L/R**	**BA**	**Coordinates (MNI)**			**T-scores**
					***x***	***y***	***z***	
Upper limb motor imagery		**HC vs. SCI**						
	49	Middle frontal gyrus	L	11	−27	42	−9	4.88
	108	Anterior cingulate	L	32	−9	45	3	3.06
		Medial frontal gyrus	L	10	−6	63	21	2.32
		**SCI vs. HC**						
	74	Inferior parietal gyrus	L	40	−63	−24	21	4.70
	90	Globus pallidus	R		21	−12	3	4.18
		Thalamus	R		15	−30	0	3.98
	93	Precentral gyrus	L	4	−15	−30	72	2.97
		Postcentral gyrus	L	2	−42	−39	63	2.84
	37	Superior temporal gyrus	R	42	66	−33	18	2.77
	42	Inferior parietal gyrus	R	40	42	−51	57	2.65
	39	Precentral gyrus	R	6	42	−3	42	2.54
Lower limb motor imagery		**HC vs. SCI**						
	114	Anterior cingulate	R	24	3	36	0	3.72
		Medial frontal gyrus	R	10	3	54	3	2.71
	43	Superior temporal gyrus	R	22	63	−27	3	3.45
	34	Superior frontal gyrus	R	10	30	66	6	3.18
		**SCI vs. HC**						
	34	Globus pallidus	R		24	−15	−6	5.42
	30	Lingual gyrus	L	18	−12	−67	−2	2.74

*SCI, spinal cord injury; HC, healthy control; BA, brodmann areas; MNI, Montreal Neurological Institute; L, left; R, right*.

Similarly, two group contrasts were conducted for the BOLD responses elicited during the lower-limb condition. For the HC vs. SCI group contrast, the HC group showed significantly stronger BOLD responses than the SCI group in the right ACG, right MeFG, right STG, and right superior frontal gyrus (SFG) (Table 4, Figure 2B). For the SCI vs. HC contrast, the SCI group showed significantly stronger BOLD responses than the HC group in the right GP and left lingual gyrus (LG). According to MNI coordinates, the BOLD responses clustered in the GPe.

### Brain Imaging–Whole-Brain Analysis Between Two Different Tasks

**Figure 3:**
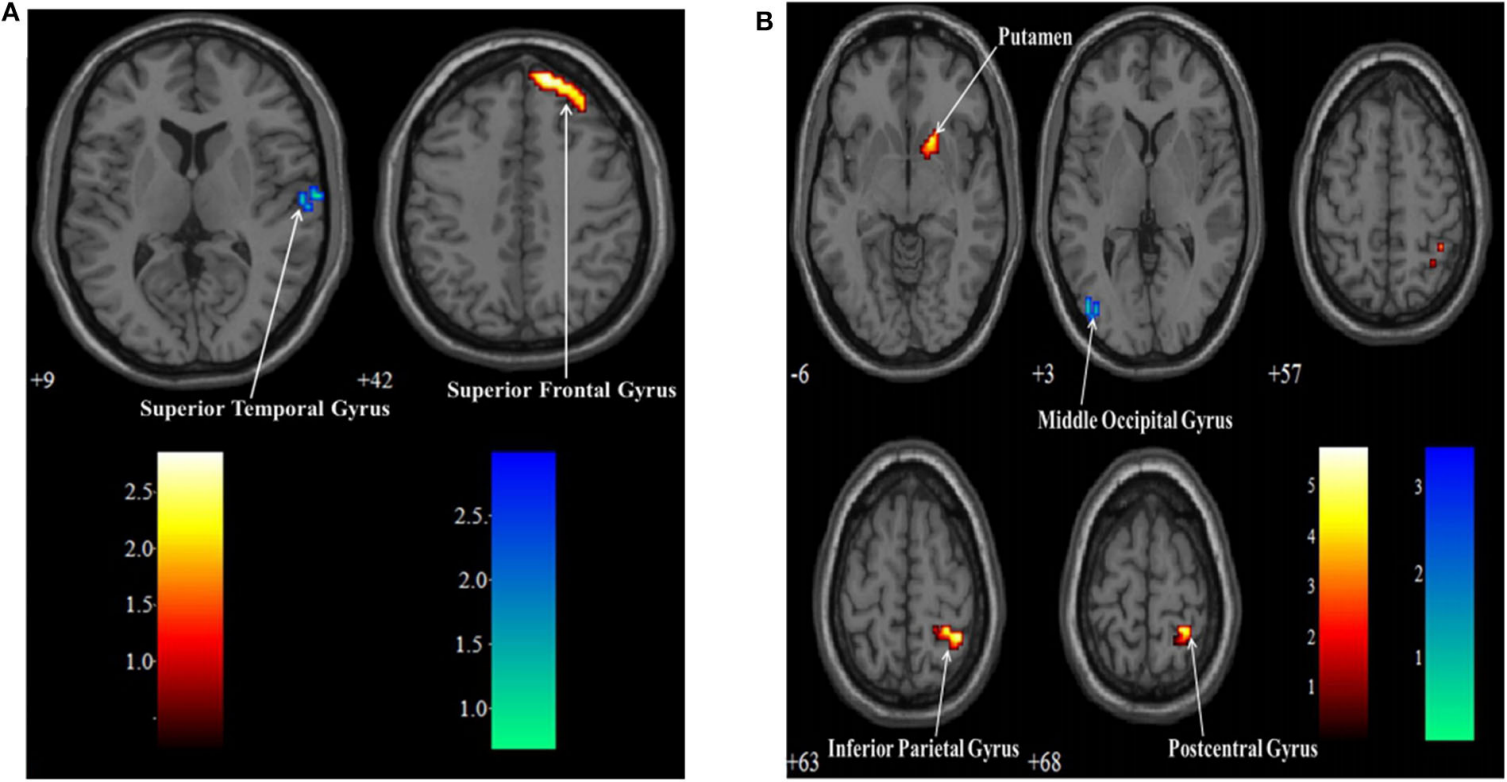
Contrasts of BOLD responses between upper and lower limb motor imagery tasks in the SCI group **(A)** and HC group **(B)**. Warm color: Upper limb vs. Lower limb; Cool color: Lower limb vs. Upper limb.

There were two MI task contrasts conducted for the BOLD responses elicited in two groups. In the SCI group, when the lower MI was compared to upper MI, the lower MI task showed stronger BOLD responses than the upper MI in the left middle occipital gyrus (MOG). When the upper MI was compared to lower MI, the upper MI had stronger BOLD responses than the lower MI in the right PosCG, IPG, and putamen (Table 5, Figure 3A). In the HC group, when the lower MI was compared to upper MI, the lower MI task showed stronger BOLD responses than the upper MI in the left STG. When the upper MI was compared to lower MI, the upper MI had stronger BOLD responses than the lower MI in the right SFG (Table 5, Figure 3B).

**Table 5:** Contrasts of BOLD responses between upper and Lower limb motor imagery tasks in the SCI and HC groups.

**Groups**	**Cluster**	**Label**	**L/R**	**BA**	**Coordinates(MNI)**			**T-scores**
					***x***	***y***	***z***	
SCI group		**Lower vs. Upper**						
	24	Middle occipital gyrus	L	19	−45	−78	3	3.43
		**Upper vs. Lower**						
	51	Postcentral gyrus	R	5	27	−38	63	5.80
		Inferior parietal gyrus	R	40	39	−41	57	5.37
	23	Putamen	R		18	14	−6	5.13
HC group		**Lower vs. Upper**						
	20	Superior temporal gyrus	L	42	69	−12	9	2.31
		**Upper vs. Lower**						
	60	Superior frontal gyrus	R	8	15	51	42	2.63

*SCI, spinal cord injury; HC, healthy control; BA, brodmann areas; MNI, Montreal Neurological Institute; L, left; R, right*.

### Brain Imaging–Regional Time-Course Analysis of the BOLD Responses

The regional time-course analysis was based on the regions activated in the whole-brain analysis. The regions contained the neural substrates that showed significant between-group contrasts in BOLD responses for both the upper-limb and lower-limb conditions and the neural substrates obtained from the significant between-condition contrasts for the SCI and HC groups. The percentage changes in the MR signals across seven time points (2 s for each time point) were tested for between-group differences for each identified region. A repeated-measure ANOVA of Group (SCI vs. HC) × Time (one-seven time points) was conducted. The same procedure was repeated for the upper-limb and lower-limb conditions. In this study, ROIs were defined with reference to the neural substrates, which showed a significant Group × Time interaction on the MR signal changes across the first 12 s in the between-group contrasts of BOLD responses for the upper-limb and lower-limb conditions. Further analyses of the comparisons of the MR signal changes in the time points with maximum differences were carried out by mixed-effect models for the ROIs (Table 6).

**Figure 4:**
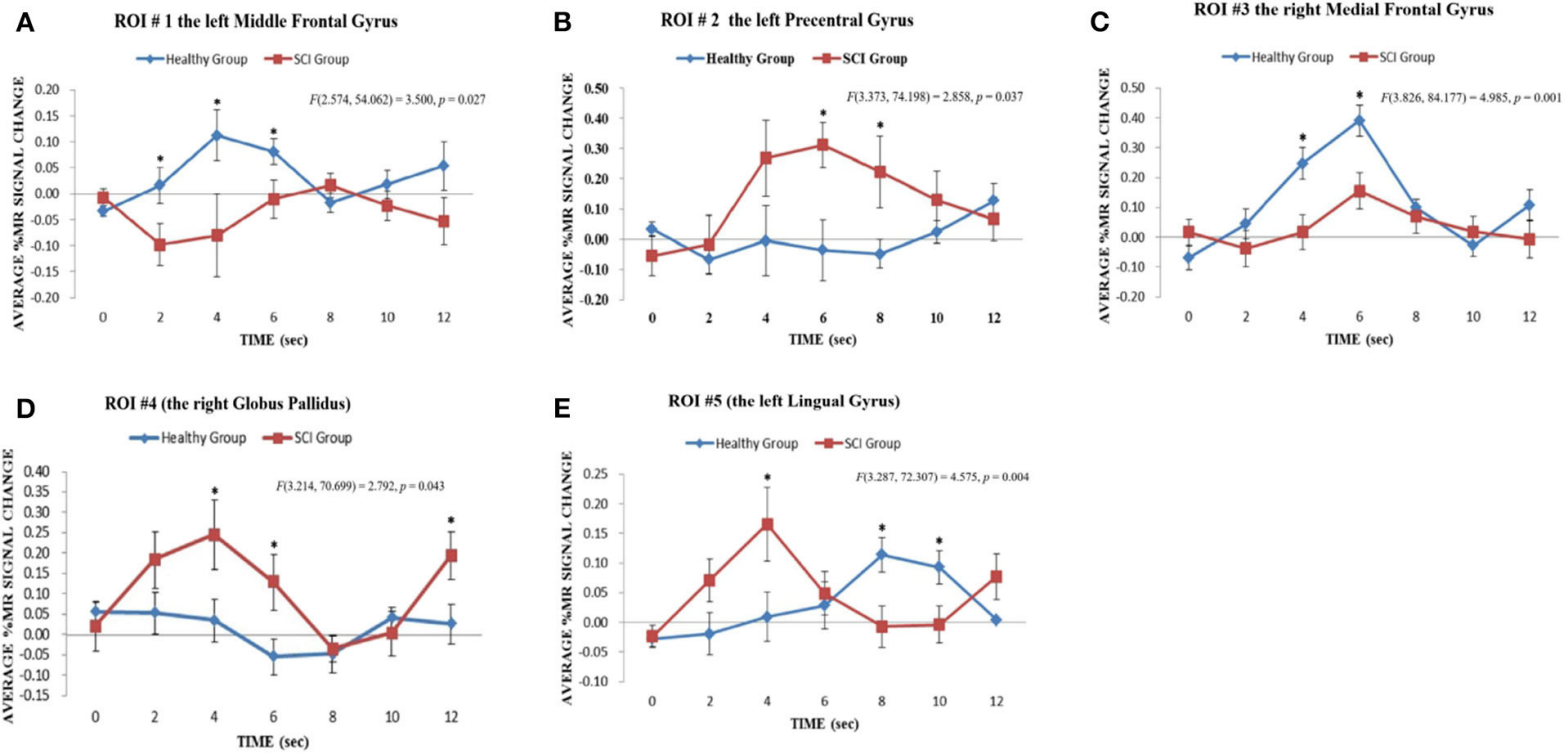
Results of regional time course analyses for ROIs. The regional time course analyses for ROI #1, ROI #2, and ROI #3 revealed the significant interaction effect between Group x Time on the average % MR signal changes elicited in the upper limb motor imagery task across time points 0–12 s in the contrast of the HC group vs. the SCI group: **(A)** ROI #1, the left middle frontal gyrus [*F*_(2.574, 54.062)_ = 3.500, *p* = 0.027]; **(B)** ROI #2, the left precentral gyrus [*F*_(3.373, 74.198)_ = 2.858, *p* = 0.037] and **(C)** ROI #3, the right medial frontal gyrus [*F*_(3.826, 84.177)_ = 4.985, *p* = 0.001]. *p < 0.05. The regional time course analyses for ROI #4 and ROI #5 revealed significant interaction effects between Group x Time on the average % MR signal changes elicited in the lower limb motor imagery task across time points 0–12 s in the contrast of the SCI group vs. the HC group: **(D)** ROI #4, right globus pallidus [*F*_(3.214, 70.699)_ = 2.792, *p* = 0.043]; **(E)** ROI #5, left lingual gyrus [*F*_(3.287, 72.307)_ = 4.575, *p* = 0.004]. *p < 0.05.

For the upper-limb condition, in the HC group vs. the SCI group, the left MeFG was defined as ROI #1 [*F*_(2.574, 54.062)_ = 3.50, *p* = 0.03] (Figure 4A). In the SCI group vs. the HC group, the left preCG was defined as ROI #2 [*F*_(3.373, 74.198)_ = 2.86, *p* = 0.04] (Figure 4B). For the lower-limb condition, the right MeFG was defined as ROI #3 [*F*_(3.826, 84.177)_ = 4.99, *p* = 0.001] in the HC group vs. the SCI group (Figure 4C). The right GP [*F*_(3.214, 70.699)_ = 2.79, *p* = 0.04] and the left LG [*F*_(3.287, 72.307)_ = 4.58, *p* = 0.004] were defined as ROI #4 (Figure 4D) and ROI #5 (Figure 4E), respectively, in the SCI group vs. the HC group.

**Table 6:** Comparisons of the maximum difference value of the average % MR signal change of the ROIs.

**ROI**	**L/R**	**Label**	**Time points with significant maximum difference**	**Average % MR signal change (SEE)**				***t*-values**	***p*-values**
				**SCI group**		**HC group**			
				**Upper limb**	**Lower limb**	**Upper limb**	**Lower limb**		
1	L	Middle frontal gyrus	2s	−0.098 (0.041)	–	0.016 (0.034)	–	2.154	0.043
			4s	−0.080 (0.079)	–	0.112 (0.049)	–	2.163	0.042
			6s	−0.010 (0.037)	–	0.081 (0.025)	–	2.109	0.047
2	L	Precentral gyrus	6s	0.313 (0.074)		−0.036 (0.100)		−2.708	0.013
			8s	0.224 (0.119)		−0.049 (0.048)		−2.254	0.034
3	R	Medial frontal gyrus	4s	–	0.017 (0.059)	–	0.247 (0.053)	2.921	0.008
			6s	–	0.156 (0.062)	–	0.391 (0.052)	2.928	0.008
4	R	Globus pallidus	4s	–	0.035 (0.086)	–	0.244 (0.052)	2.166	0.041
			6s	–	−0.055 (0.069)	–	0.129 (0.044)	2.303	0.031
			12s	–	0.026 (0.059)	–	0.194 (0.049)	2.212	0.038
5	L	Lingual gyrus	4s	–	0.166 (0.062)	–	0.009 (0.038)	−2.226	0.037
			8s	–	−0.007 (0.034)	–	0.114 (0.044)	2.093	0.048
			10s		−0.004 (0.031)	–	0.093 (0.033)	2.131	0.044

*SCI, spinal cord injury; HC, healthy control; ROI, region of interest; L, left; R, right*.

### Correlation Analysis

**Figure 5:**
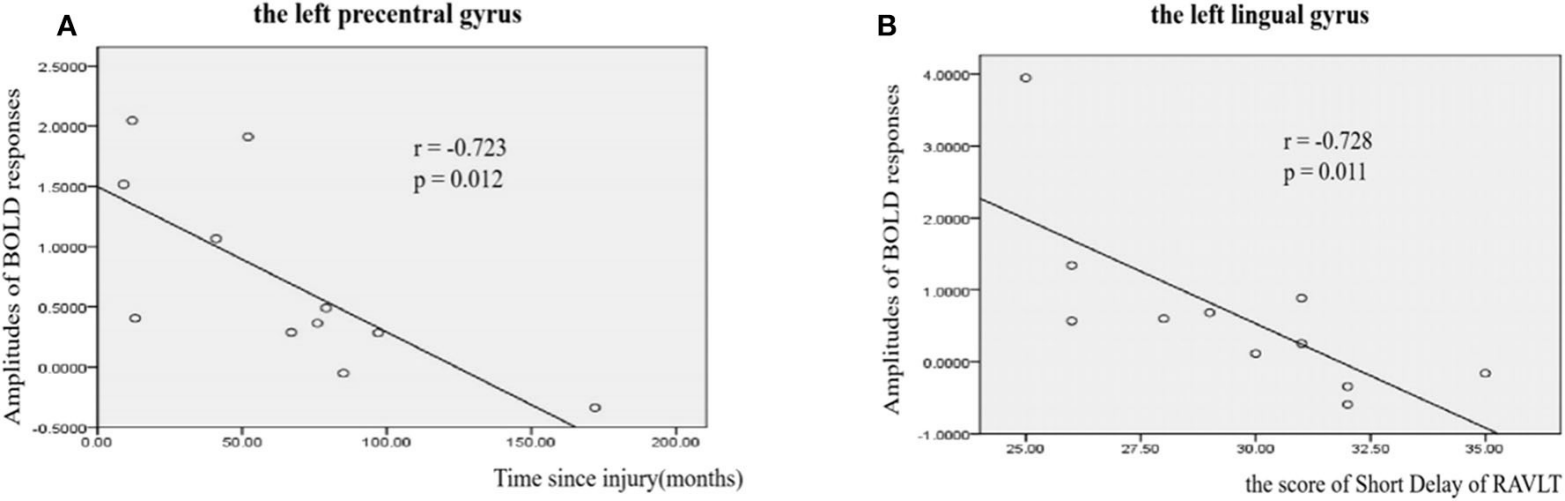
Correlations between amplitudes of BOLD responses and clinical measures in SCI patients. **(A)** Correlations between amplitudes of BOLD responses and time since injury in left precentral gyrus (*r* = −0.723, *p* = 0.012). **(B)** Correlations between amplitudes of BOLD responses and the score of Short Delay of RAVLT in left lingual gyrus (*r* = −0.7238, *p* = 0.011).

A correlation analysis was conducted for the BOLD responses of these ROIs, the behavioral measures, and the clinical measures with significant differences. The results showed that for the upper-limb condition in the SCI group, the amplitudes of BOLD responses in the left preCG (ROI #2) were negatively correlated with the time since injury (*r* = −0.72, *p* = 0.012) (Figure 5A). For the lower-limb condition in the SCI group, the amplitudes of BOLD responses in the left LG (ROI #5) were negatively correlated with the scores on the Short Delay task of the RAVLT (*r* = −0.73, *p* = 0.011) (Figure 5B).

## Discussion

In this study, we investigated the functional reorganization in complete thoracic SCI patients. During upper and lower MI, SCI patients showed significantly stronger BOLD responses in cortical and subcortical regions than the HCs. Meanwhile, differences in brain activation were observed between upper and lower MI in patients with SCI. Moreover, the amplitudes of BOLD responses in left preCG and left LG were negatively correlated with the time since injury and the scores of Short Delay of RAVLT, respectively. These results suggested that brain plastic changes occur in upper and lower MI in patients with complete thoracic SCI, which might be suggestive of compensatory mechanisms due to the loss of physical movement and sensory feedback in SCI patients.

Our results also showed between-group differences in the BOLD signals both in upper- and lower-limb MI. The HC group had stronger BOLD responses in frontal gyrus (MiFG, SFG, and MeFG) than the SCI group. The left MiFG, which was defined as ROI #1, is involved in the performance of the executive function and emotion regulation (18–22). The SFG has been reported to be involved in various cognitive and motor control tasks (23). The MeFG is a region associated with high-level executive functions and decision-related processes (24). These regions are involved in executive and cognitive processes. Our findings of functional abnormalities in the frontal area in patients may be linked to the sensorimotor deficits and mental state after SCI. The right ACG and the right STG were also found to be activated in whole-brain analysis, which concurs with previous studies (25, 26), on motor programming for locomotion. This frontal–cingulate–temporal network was found to mediate the working memory related to MI processing (25, 26). Furthermore, we found decreased activation in ACG, the cognitive-related region, during both the upper- and lower-limb tasks in SCI patients, which is consistent with previous studies (27, 28). Meanwhile, cognition evaluation (RAVCT and TMT) was lower in SCI patients than in HCs. Craig et al. (29) reported cognitive impairment and negative mood states in patients with SCI. Decreased activation in ACG might be related to the decreased cognition. Thus, future studies should focus more on cognitive performance in these populations.

Compared with HCs, SCI patients showed stronger BOLD signals in cortical and subcortical regions involved in the sensorimotor and audio–visual function. Increased activation in sensorimotor areas was previously reported in patients with SCI when compared to the HCs (7, 10). This study further confirmed that audio–visual regions were activated in SCI patients during MI. During upper-limb MI, increased activation was found in posCG, preCG, thalamus, GPe, STG, and IPG in SCI patients; PosCG and preCG are classical sensorimotor areas, which are important for sensory and motor signal processing. The thalamus is considered a relay center in the basal ganglia–thalamocortical circuit, which mediates motor control processes (30). This sensorimotor activation could be caused by increased use of upper limbs in SCI patients who had paralysis of the lower limbs (14). Furthermore, GPe, STG, and IPG are regions associated with visual and auditory information processing (31). It indicated that audio–visual areas are activated in SCI patients during upper limbs MI, thus suggesting that such systemic changes are not restricted to the affected body parts—the lower limbs—but to the upper limbs as well.

Stronger activation in GPe (ROI #4) and LG (ROI #5) were observed among SCI patients when performing a lower MI task. For the SCI group, a greater percentage signal change in the BOLD responses was observed during the first half of the foot imagery task (Figure 4D), suggesting a possible involvement of the right GPe in the early stage of MI. Anatomically, the GPe serves as a relay station connecting the cerebral cortex, thalamus, cerebellum, and spinal cord (32–34). During motor control, the GP mediates force regulation (35), speed of cognitive processing (36), and sensory gating of motor control (36). Compared to the internal part of the GP (GPi), the GPe was more a control station for the excitability of the cortical–basal ganglia–thalamus network in movement (37) and the cortical–basal ganglia–cerebellar network for motor memory storage (38). In Albin's study (39), the GPe responded to motor-related memory while GPi did not. The activation of GPe in lower-limb MI suggested increases in mediated retrieval of motor images for image generation.

Another essential activation was located in the left LG (ROI #5), and significant between-group differences in the regional time course analysis were revealed at 4, 8, and 10 s (Figure 4E, Table 6). The percentage of changes was different between the first and second halves of the responses. The first half was dominated by the larger percentage of BOLD signal change among the SCI patients in the left LG. In contrast, the latter half of the responses was dominated by the larger percentage of change among HCs. The initiation of the responses in the left LG in SCI patients appeared to be earlier than that of HCs. The involvement of the left LG in the imagery of lower-limb MI among SCI patients is a new finding. It is known that the LG mediates visual perception and visual-MI process (40, 41). Stronger activation in left LG, which is located in the occipital lobe, suggested that SCI patients relied more on the visual system to mediate MI of the foot. Meanwhile, we also found stronger BOLD responses in middle occipital gyrus in lower-limb MI than upper-limb MI in SCI patients. Previous studies concluded that the occipital lobe had a vital role in visuomotor imagery by mediating image retrieval and generation in working memory during visual–motor imagery processing (40, 42, 43). Our findings indicated that SCI patients used the visual system to generate, maintain, and transform lower-limb motor images during visualization of the related movements. The SCI patients might develop visual compensation for lacking sensory input from the lower limbs. This could be attributed to the long-term lack of sensory input from the paralyzed lower limbs due to SCI.

The cognitive measures showed poor performances on the Short Delay and Recognition scores on the RAVLT and the difference scores on the TMT (B minus A), thus suggesting the cognitive declined in SCI patients. This finding was consistent with the study conducted by Jegede et al. (17). The cognitive function mainly relied on processing speed, memory recall, inhibition, and set-shifting. MI involves a series of mental processes, including image generation, image maintenance, image inspection, image transformation, and motor preparation and programming (40, 44, 45). There is ample evidence that the control of any voluntary movement relies on both higher-level cognitive and lower-level movement mechanisms (40, 44, 45). Cognitive control supports flexible behavior by selecting actions that are consistent with the goals and appropriate to the environment (46). For instance, even a simple key-press choice reaction time task requires a cognitive component that is related to action selection and is dependent on the premotor cortex (47). The poor cognitive performance in SCI patients may result from a deficit in cognitive output commands that rely on feedback information received from the outside. Furthermore, the HCs had significantly stronger BOLD responses than the SCI patients in the frontal areas and the left ACG for both upper and lower-limb MI. The frontal areas and ACG are sensitive to control demands (46, 48). Our study showed that the HCs had increased activation in ACG than SCI patients, which might be related to the poor performances on recognition. Zhu et al. (49) found decreased brain regional homogeneity (ReHo), reflecting the temporal homogeneity of the BOLD signal associated with cognitive control, such as the bilateral dorsal ACG.

Our study showed that for the upper-limb MI, the amplitudes of BOLD responses in the left preCG were negatively correlated with the time since injury. The activation of the upper-limb innervation area of the MI decreased as the time from the injury passed on. The changes could be a consequence of the injuries to the spinal cord suffered by SCI. For the lower-limb MI in the SCI group, the amplitudes of BOLD responses in the left LG were negatively correlated with the Short Delay scores on the RAVLT. The LG represents part of the primary visual cortex and receives direct input from the ipsilateral lateral geniculate nucleus (49). Greater activation in the left LG may indicate greater visual functional participation in MI.

## Limitations

Several limitations should be addressed in the present study. First, the small sample size in the two groups diminished the statistical power. Second, the study explored functional changes in SCI patients with a broad range of disease duration.

## Conclusion

This study investigated the functional reorganization of the brains in individuals suffering from SCI. The HCs revealed stronger BOLD responses in frontal gyrus and ACC than the SCI patients in MI. In contrast, the SCI patients showed stronger activation in the right GPe and left LG than the HCs during imagery of repetitive movements of the lower limbs. These changes were more likely affected by the consequences of paralysis of the lower limbs due to SCI.

## Data Availability Statement

All datasets generated for this study are included in the article/supplementary material.

## Ethics Statement

The procedures in this study were vetted and approved by the Ethics Committee on Human Studies of CRRC and Departmental Ethics Committee of Department of Rehabilitation Sciences, the Hong Kong Polytechnic University. The patients/participants provided their written informed consent to participate in this study. Written informed consent was obtained from the individual(s) for the publication of any potentially identifiable images or data included in this article.

## Author Contributions

JiL, CC, FG, and YG conceived the study and designed the experiments. MY, LD, and DY helped in designing the experiments for better performance. FG and YG performed the experiments, wrote the manuscript, performed data preprocessing, and statistical analysis. WY and ZC performed MRI scanning. All authors have read and approved the final manuscript.

## Conflict of Interest

The authors declare that the research was conducted in the absence of any commercial or financial relationships that could be construed as a potential conflict of interest.
